# Assessing the sustainability of the homestay industry for the East Coast of Malaysia using the Delphi approach

**DOI:** 10.1016/j.heliyon.2023.e21433

**Published:** 2023-10-31

**Authors:** Fatin Amira Zamzuki, Muhamad Safiih Lola, Elayaraja Aruchunan, Mohana Sundaram Muthuvalu, Ribed Vianneca W. Jubilee, Nurul Hila Zainuddin, Abdul Aziz K. Abdul Hamid, Nor Aieni Mokhtar, Mohd Tajuddin Abdullah

**Affiliations:** a Faculty of Ocean Engineering Technology and Informatics, Universiti Malaysia Terengganu, 21030, Kuala Nerus, Terengganu, Malaysia; b Special Interest Group on Modeling and Data Analytics (SIGMDA), Universiti Malaysia Terengganu, 21030, Kuala Nerus, Terengganu, Malaysia; c Institute of Mathematical Sciences, Faculty of Science, University of Malaya, 50603, Kuala Lumpur, Kuala Lumpur Federal Territory, Malaysia; d Department of Fundamental and Applied Sciences, Universiti Teknologi PETRONAS, 32610, Seri Iskandar, Perak Darul Ridzuan, Malaysia; e Labuan Faculty of International Finance, Universiti Malaysia Sabah Labuan International Campus, Labuan Federal Territory, 87000, Malaysia; f Mathematics Department, Faculty of Science and Mathematics, Universiti Pendidikan Sultan Idris, 53900, Tanjong Malim, Perak Darul Ridzuan, Malaysia; g Special Interest Group on Applied Informatics and Intelligent Applications (AINIA), Universiti Malaysia Terengganu, 21030, Kuala Nerus, Terengganu, Malaysia; h Institute of Oceanography and Environment, Universiti Malaysia Terengganu, 21030, Kuala Nerus, Terengganu, Malaysia; i Academy of Sciences Malaysia, Menara Matrade, Tingkat 20, Sayap Barat, Jalan Sultan Haji Ahmad Shah, 50480, Kuala Lumpur Federal Territory, Malaysia

**Keywords:** Homestay management, Sustainability, Ecotourism, Delphi approach, COVID-19, Tourism

## Abstract

Homestay ecotourism in Malaysia has been extensively examined in terms of its concepts, approaches, activities, and community engagement. However, a comprehensive assessment of the sustainability factors pertaining to host families remains a critical area awaiting exploration. This is paramount for ensuring the long-term viability of homestays and fostering economic benefits within rural communities. The present study seeks to establish direct subjective measurements for evaluating the interplay between local communities, tourism, and resources in safeguarding sustainable homestays. Utilizing the Delphi approach, this research conducted interviews with 51 experts who were actively involved in six homestays located on the East Coast of Peninsular Malaysia. The objective was to identify key evaluation indicators pertinent to the homestay industry. The findings underscored the pivotal roles played by community resources and tourism in the sustainability of homestays. Additionally, environmental, economic, and social factors emerged as crucial components for maintaining the industry's sustainability. This innovative assessment methodology offers a valuable instrument for enhancing the sustainability of the homestay sector, especially in the wake of the COVID-19 pandemic. By embracing this approach, homestay operators can fortify their sustainable management practices and prepare themselves for future pandemics. This study represents a significant contribution to the field of homestay ecotourism, emphasizing the imperative for continued research in this dynamic domain.

## 1 Introduction

Malaysia, celebrated for its rich biodiversity, multicultural tapestry, and captivating historical sites, also distinguishes itself through a diverse array of rural tourism experiences and destinations. Notably, homestays occupy a prominent position among these rural tourism offerings. In the Malaysian context, these homestays are referred to as “*Inap Desa*”, a program initiated by the Ministry of Tourism, Arts, and Culture [1]. During the Ninth Malaysia Plan (2006–2010), homestays emerged as a pivotal component of the country's rural development strategy, aimed at alleviating poverty within the lower 40% income bracket and middle-income groups [2]. Homestays present a multifaceted approach to income generation, encompassing service charges, user fees, collaborative ventures with the private sector, and contributions to the local economy. Furthermore, the expansion of homestay initiatives brings forth new employment opportunities, creating roles such as instructors, administrators, analysts, and officers to meet the burgeoning demands within the dynamic tourism sector.

The inception of ‘*Inap Desa*’ in Malaysia has its origins in the story of a local resident named Mak Long, who resided in the charming village of Cherating. Back in the early 1970s, Mak Long opened her own home to provide accommodations, breakfast, and dinner to a group of wandering tourists [3]. In essence, homestays refer to local residences that extend an invitation to travelers, offering them the opportunity to stay with a host family in exchange for a fee. These accommodations typically feature budget-friendly amenities [4]. Guests find themselves in close proximity to the host family, sharing both private facilities and communal spaces. This arrangement creates a unique opportunity for tourists to immerse themselves in the local culture and traditions, fostering cultural exchange and understanding. Engaging in these experiences and sharing insights into Malaysian culture serve as incentives for local communities participating in the *‘Inap Desa’* program to reap economic benefits. Positioned as a sustainable tourism approach, homestays are expected to strike a harmonious balance between preserving traditional practices and promoting sustainable development.

Recognizing the substantial potential for rural economic growth, the Ministry of Tourism, Arts, and Culture (MOTAC) has undertaken the responsibility of elevating and promoting the *‘Inap Desa’* program as a cornerstone of rural tourism initiatives. A pivotal moment in this endeavor was the formulation of the Rural Tourism Master Plan in 2001, which aimed to position the *‘Inap Desa’* program as a driving force behind the development of rural communities. This initiative was subsequently incorporated into the 9th Malaysia Plan (2006–2010). According to MOTAC's report [5], as of December 2019, the *‘Inap Desa*’ cluster had successfully registered 219 houses, engaging a total of 4210 participants and offering a combined room count of 5956. This collective effort translated into a remarkable cumulative income of RM29,662,211.60 (equivalent to USD6,525,686.55; RM1 = USD0.22).

These dedicated efforts bore fruit when the Malaysian Homestay programs received international recognition, earning an esteemed award from the United Nations World Tourism Organization [6] in the Rural Area Tourism category. This prestigious accolade was in recognition of the impressive growth in the number of tourists participating in homestay programs, which saw a remarkable 32% increase, surging from 161,561 guests in 2009 to 213,263 guests just three years later. While the homestay industry has proven highly successful in generating significant economic benefits for rural communities, it has also encountered challenges in sustaining its long-term objectives.

Understanding the intricate interplay of extrinsic and intrinsic factors that impact the sustainability of homestays is imperative for making well-informed decisions and taking the necessary actions to ensure the continued vitality of the homestay industry.

The “Visit Truly Asia Malaysia 2020" campaign had set ambitious targets, envisioning the arrival of 30 million tourists in Malaysia and the generation of RM100 billion (USD22 billion) in revenue for the year 2020. Unfortunately, the campaign had to be abruptly canceled as international borders were sealed to combat the spread of the COVID-19 virus. This unforeseen situation led to staggering losses in the travel and tourism sector, with an estimated value decline of approximately USD 4.5–4.7 trillion, resulting in a dramatic 49.1% drop in GDP [7]. Globally, this translated into a staggering loss of 62 million jobs, reflecting an 18.5% decrease in 2020.

In line with these significant global disruptions, Tourism Malaysia reports a drastic decline in international tourist arrivals, plummeting from 26,100,784 in 2019 to a mere 4,332,722 in 2020, representing a staggering decrease of over 83% [8]. Concurrently, tourist revenue witnessed an 85% plunge, dwindling from RM86.14 billion in 2019 to RM12.69 billion in 2020 (equivalent to USD2.68 billion). Prior to these challenging circumstances, tourism held the distinction of being the third most substantial contributor to GDP of Malaysia, following manufacturing and commodities.

Therefore, it is imperative to embark on a comprehensive exploration encompassing all facets of ecotourism and their implications for the enduring sustainability of homestays. As noted by Ross and Wall [9], a standardized set of evaluation criteria for assessing the state of ecotourism at specific locations is yet to be fully developed. Similarly, within the context of homestays, the intricate interplay among various factors complicates efforts to gauge the industry's sustainability effectively.

Moreover, there is a notable scarcity of environmental assessments and audits conducted on numerous homestays and well-known tourist destinations. Similarly, in the realm of ecotourism, the sustainability of this industry hinges on a multifaceted array of factors, encompassing the availability of resources, active community engagement, and the diversity of tourism products [9,10].

In order to comprehend the interrelation among these factors that contribute to the continued competitiveness of homestays, it is imperative to establish rational metrics for evaluating their sustainability. These quantifiable benchmarks can be categorized into “objective” or “subjective” criteria. Objective indicators, also known as extrinsic indicators, pertain to quantitative data, often expressed through mathematical equations. These indicators have gained widespread acceptance due to their perceived robustness. Conversely, subjective or intrinsic indicators are of a qualitative nature, delving into personal emotions and viewpoints. While not all sustainability indicators are readily measurable, some may necessitate a degree of subjectivity [11]. However, this limitation does not diminish their value as informative tools for sustainable management enhancement.

Previous research efforts in the realm of homestays have predominantly focused on areas encompassing socio-cultural sustainability, rural community-based tourism, and the establishment of community-oriented rural homestay experiences [12]. Nevertheless, within the Malaysian context, there exists a notable gap in the literature when it comes to evaluating the sustainability of homestays using the Delphi approach. This study's principal objective is to construct direct subjective measurements aimed at assessing the sustainability of the homestay industry in the East Coast states of Peninsular Malaysia, employing this innovative evaluation method to gauge the sector's sustainability.

The utilization of predictive models through the Delphi method represents a pioneering approach for evaluating the sustainability of homestays within the broader tourism sector. Within the context of homestays, sustainable development can be defined as the endeavor to maintain the capacity of natural ecosystems to strike a balance between the natural resources and ecosystem services upon which the economy and society rely. This aligns with the established principles guiding the attainment of the Sustainable Development Goals for 2030 (SDG 2030).

The exploration of homestays closely aligns with several Sustainable Development Goals (SDGs) covering various dimensions, including social, economic, and environmental aspects. These encompass SDG 1: No Poverty, SDG 8: Decent Work and Economic Growth, SDG 11: Sustainable Cities and Communities, SDG 12: Responsible Consumption and Production, and SDG 13: Climate Action. By addressing these SDGs, this study underscores the potential of homestays as a sustainable tourism option that can contribute significantly to the overarching objectives of the SDG 2030 agenda. It emphasizes the importance of considering social, economic, and environmental factors as integral components in promoting sustainable development within the context of homestay tourism.

Recognizing the paramount significance of comprehending these factors in relation to the sustainability of homestays, this study endeavors to employ subjective measures in a direct inquiry into the perceptions held by stakeholders regarding resources, community dynamics, and tourism opportunities. These subjective insights form the foundation for our assessment of sustainability. As such, we have formulated the following three research questions (RQ) to systematically evaluate the sustainability of the homestay industry in Malaysia.

RQ1 Does the study provide sufficient evidence to support the potential for sustainable homestays in the future?

RQ2 What are the key factors influencing the sustainability of homestays?

RQ3 Are there alternative approaches to enhance the sustainability of homestays without compromising the natural ecosystem?

The subsequent sections of this paper are structured as follows: Section 2 offers an extensive review of the literature on homestay industry sustainability. Section 3 provides an intricate explanation of the proposed theoretical framework. Section 4 delineates the sustainability evaluation framework in detail. In Section 5, we delve into the methodology employed for assessing the sustainability of the homestay industry. Section 6 delves into a comprehensive discussion of the empirical results obtained from our evaluation of homestay sustainability. Lastly, Section 7 provides a succinct summary of the paper, highlights research limitations, and outlines potential avenues for future research.

## 2 Literature review

### 2.1 Homestay and sustainable tourism

Homestays represent a promising product with substantial potential for advancing the sustainability of both the natural environment and local communities within the tourism industry [13,14]. They provide operators with an avenue to explore innovative platforms within the tourism sector, fostering an appreciation for and engagement with the societal well-being, customs, and traditions prevalent in the host villages [15]. From a conceptual standpoint, hosting homestays can significantly improve the quality of life for the hosts, as it offers them the opportunity to bolster their economic well-being through the income generated from facility rentals [12]. Concurrently, these homestays serve as a conduit for presenting the rich tapestry of traditional culture and knowledge practiced by the local community to visiting tourists [16].

In Malaysia, the registered *‘Inap Desa’* program, established in the 1980s, is specifically geared towards enhancing the income of rural residents by introducing novel economic endeavors within the tourism sector [12]. By actively promoting homestay tourism, the government seeks to advance its agenda of generating employment opportunities and alleviating poverty within the participating communities [17]. In a broader context, ‘Inap Desa’ or homestay initiatives hold immense potential for development, primarily due to their established reputation, which can wield a substantial positive impact on the community, particularly in terms of income generation [12,18]. This approach stands out as the most effective means of assisting middle-income families, especially those in rural areas, in augmenting their earnings, while also making a commendable contribution to the local economy by bolstering financial benefits.

One of the primary draws for both local and international tourists lies in the distinctive features and experiences that await visitors. The homestay industry plays a pivotal role in enabling local residents to establish a consistent source of income for their sustenance [19,20]. Tourism contributes significantly to enhancing the quality of life, particularly for rural inhabitants, as it positively impacts various socio-economic factors while also preserving the natural environment [21]. Homestays emerge as an appealing and sustainable rural tourism offering, fostering local employment opportunities, safeguarding the environment, and preserving the richness of cultural heritage [22,23].

### 2.2 Homestay and sustainability development

Over the past few decades, the rapid expansion of the tourism industry has given rise to the concept of sustainable homestay tourism, a topic extensively explored in prior research [[30], [31], [32]]. This concept offers visitors a unique opportunity to engage with rural communities, fostering cultural exchange and providing them with valuable insights into local ways of life and traditions [33]. Sustainable development serves as a foundational conceptual framework for community and social advancement, characterized by a long-term perspective on resource utilization and closely aligned with environmentally responsible tourism [31,34].

The sustainability of homestays is rooted in the fundamental principles of sustainable development, which encompass three core pillars: resources (environment), community (local residents and communities), and economics (tourists) [10,32,35,36]. Sustainable development is a commonly employed term, particularly in the context of tourism-dependent communities, and it pertains to the “triple bottom line” approach, where policies and actions are designed to balance social, economic, and environmental costs and benefits [32,37]. This approach is also among the most prevalent strategies for enhancing societal resilience [25].

Community development strategies show a profound respect for the natural environment. They have been demonstrated to hold significant promise in promoting biodiversity due to their integration of ecotourism activities [9,30], underscored by a firm dedication to conserving biodiversity [31,38]. In addition, it uses natural resources and local culture to provide tourists with local experiences [10,30,35]. It also provides sports, recreation, and other activities that allow visitors to fully enjoy their leisure time [15,16,18,39].

### 2.3 Homestay and methods for sustainability

In order to ensure the continued prosperity and resilience of homestays, a range of community development approaches has gained widespread adoption, including Community-Based Tourism (CBT) [12,18], Community-Based Ecotourism (CBET) [24], Community-Based Tourism Standards (CBTS) [25], and Community Agrotourism (CBAT) [26]. These methodologies are specifically designed to enhance the capacity of rural communities in the planning and management of tourism resources, all the while ensuring active involvement from the local community [12,[27], [28], [29]].

In the studies conducted by Refs. [12,18,[24], [25], [26], [27], [28], [29]], a combination of both qualitative and quantitative methodologies was employed to evaluate the immediate impacts of tourist arrivals on the economy and society. Researchers also ventured into mixed-method analyses, encompassing aspects related to the community, economy, and Community-Based Tourism (CBT) [25,39]. Furthermore, research efforts included exploratory investigations, conceptual paper analyses, and comprehensive reviews, addressing topics such as community, economy, society, and heritage [16,40]. Additionally, investigations into the sustainability of homestays involved the application of modeling and analytical techniques grounded in Structural Equation Modeling (SEM) [17,30,31,41].

In the contemporary landscape, the influence of social media platforms like Facebook, Twitter, Telegram, and others has emerged as a pivotal factor in determining the sustainability of homestays. Numerous studies, particularly within ASEAN countries, have delved into the sustainability of homestays, with a specific focus on the role of social media. These studies have employed a variety of statistical methods to analyze this relationship. For instance, Yong and Hassan [42] explored the connection between social media marketing and entrepreneurial success in homestay businesses, utilizing Partial Least Squares (PLS) analysis. Similarly, Murniati et al. [43] conducted an investigation into the awareness of homestays from the perspective of social media as a marketing tool, employing thematic analysis within the context of Community-Based Tourism (CBT) among rural homestay operators in Malaysia.

## 3 Theoretical framework of the sustainability of the homestay industry

Over the past two decades, sustainable development has gained significant momentum [44], making the task of formulating relevant indicators increasingly complex, especially within the realms of policy and academia. Various international, national, local, public, and private entities have undertaken extensive efforts to define metrics that can effectively measure the enduring ability of both the natural world and society to coexist and thrive harmoniously. Numerous indicators have been identified as valuable tools and benchmarks for assessing and tracking progress towards sustainable development. Consequently, these indicators of sustainable development have been categorized into four distinct groups.

The first group of indicators places a strong emphasis on the environment, focusing on receptors and the environmental effects and responses. In the second group, indicators are classified based on their spatial scale, distinguishing between global, national, and local perspectives. The third group delves into the environmental medium itself, encompassing indicators related to elements like air, water, soil, and more. Finally, the fourth group of indicators centers on the dimensions of sustainability, including ecological, economic, social, and integrated aspects [45]. Given the absence of prior research on homestay sustainability assessment, this study adopts and adapts existing concepts of sustainable development to conduct its analysis.

The perspective of homestays, intricately linked with tourism, can be categorized based on its perceived impacts across three key domains: economic, social, and environmental. This classification serves as a foundation for constructing a homestay impact scale [10]. In a similar vein, this paper aims to investigate the assessments of experts regarding the sustainability of the homestay sector. To accomplish this objective, an indicator framework encompassing both economic and environmental aspects is put into practice.

**Fig. 1:**
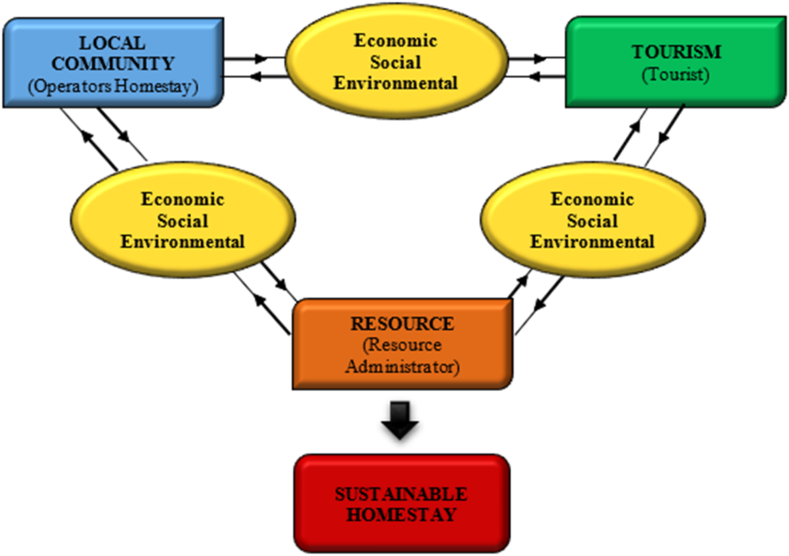
The theoretical framework for sustainable homestay. Modified from Tsaur et al. [11]. Evaluating ecotourism sustainability from the integrated perspective of resource, community and tourism. *Tourism Management,* 2006; **27**: 640–653.

The theoretical framework guiding this exploration of study of the sustainability of the homestay industry is depicted in Fig. 1, drawing upon insights from studies on ecotourism sustainability [9,10]. Within this theoretical framework, the three core elements-resources, culture, and visitors-have been prominently featured, as outlined by Ref. [11]. In line with the research by Tsaur et al. [11], it is evident that these elements exert mutual influence and engage in intricate interactions. Tsaur et al. [11] also underscore the symbiotic relationship between natural areas and local communities, further enhanced by the introduction of host families. Conversely, Ross and Wall [9,10] employ different terminology when assessing ecotourism, specifically referring to local communes, biodiversity, and tourism. Consequently, homestays are recognized as instrumental tools for both conservation and sustainable development.

In this study, a comprehensive evaluation of the sustainability of the homestay industry in the East Coast of Malaysia was undertaken, employing the Delphi approach. The research involved conducting interviews with a diverse set of stakeholders, including members of the local community, representatives from the tourism sector, and proprietors of key resources. The interview questions were thoughtfully crafted to assess perceptions and the interconnectedness of these stakeholder groups, ultimately enabling a thorough evaluation of homestay sustainability.

The initial phase of analysis concentrated on identifying the variables that naturally arose from the intricate interplay between the community, tourism, and resources, all of which collectively impact the sustainability of homestays. Subsequently, this complex network of relationships was organized into dimensions encompassing economic, societal, and environmental facets. Following this organization, the Delphi technique was applied to pinpoint pertinent evaluation indicators and allocate relative priority weights to them. This meticulous process culminated in the innovative development of the Sustainable Homestay Indicators System (SHIS).

## 4 The evaluating framework for homestays

Homestay development relies on three critical indicators: the local community, tourists, and resources, with the success of a homestay concept pivoting on the harmonious interplay among these three factors, where each contributes positively to the others [9]. It is widely recognized that changes within the economic, social, and environmental dimensions are the primary factors influencing sustainability, and these dimensions are intricately interconnected [46].

In this study, resource management, homestay operators, and tourists were selected as representative stakeholders embodying the dimensions of resources, the local community, and tourism. The significance of each stakeholder spans economic, social, and environmental aspects within the homestay context. To empirically investigate this, the study focused on homestays situated along the East Coast of Peninsular Malaysia, specifically in the states of Terengganu and Kelantan. The selection of sustainability indicators took into careful consideration the distinctive characteristics of tourist development in these particular homestays.

### 4.1 The influence of resources on the community

Homestay resources are integral in attracting tourists to these accommodations, and the sustainability of homestays is intrinsically linked to the management of these resources. Effective resource management, encompassing conservation and utilization of the surroundings near the homestay, falls under the purview of resource administration. The environment plays a pivotal role in the development of homestays, with local resources like water carrying significant importance, as exemplified in the case of Dagat Village in Lower Kinabatangan-Segama, Sabah [30].

In general, the conservation measures that resource administration can take are providing a place for agriculture, as well as observing the pollution that will occur in the homestay environment. Thus, when resources are well maintained, the quality of life of the homestay operators, staff, and other residents can be enhanced. In addition, resources also affect the community economically. This can be seen when homestay operators and staff participated in the work of the conservation of the environment which can increase their financial resources. From a social perspective, the appropriate education and training not only raise awareness in the community about environmental protection, but also support cultural resources so the population can be maintained.

### 4.2 The influence of community on resources

In the context of the homestay study in the states of Terengganu, Bachok et al. [3] noted that local community homestay operators have a significant impact on both environmental and social resources. A sudden decrease in these resources can negatively affect the quality of life of the local population and deter tourists from returning to the homestay. Therefore, resource administration should prioritize the conservation of natural resources by minimizing development in the vicinity of homestays. Excessive resource exploitation, such as logging, can jeopardize resource sustainability.

From a social perspective, the active involvement of local communities in resource conservation is crucial for maintaining the sustainability of both resources and homestays. If the local community does not uphold this foundation of homestays, it can lead to a decline in repeat visits by tourists. To promote a positive relationship with conservation efforts, local communities should have a sense of control and ownership over the planning process related to resource utilization and natural resource development.

### 4.3 The influence of resources on tourists

In terms of the environment and the economy, the tourism resources developed for homestays should possess sufficient allure to attract tourists [3]. For instance, charging fees for homestay accommodations can help offset the costs of environmental conservation efforts. On the social front, visitors come to homestays to immerse themselves in the local culture, traditions, and activities offered by the hosts. For instance, tourists often participate in activities like gardening alongside their hosts. This engagement with planting activities contributes to the restoration of natural resources.

### 4.4 The influence of tourism on resources

In terms of the environment, it's important to acknowledge that certain tourism platforms have a significant impact on the environment [11]. Therefore, it becomes imperative to establish initiatives aimed at environmental protection within the realm of tourism. These initiatives are vital for maintaining valuable resources and ultimately increasing the number of tourists visiting homestays. On the social front, homestay operators should receive high-quality environmental education. This education equips them to highlight the unique advantages of homestays to tourists. When homestay operators possess a strong foundation in environmental education, they can effectively educate tourists on responsible behavior, nurture an appreciation for nature, and contribute to the overall sustainability of the homestay.

### 4.5 The influence of the local community on tourism

From a social perspective, visitors are deeply influenced by the local culture through direct contact. Homestay owners, for example, should engage with their guests, sharing their customs and culture. This interaction not only allows tourists to immerse themselves in the local experience but also fosters an appreciation for the cultural resources and sustainability of the homestay. Consequently, it increases the likelihood of these tourists choosing to stay in a homestay again.

### 4.6 The influence of tourism on the local community

In terms of the environment, some tourists engage in activities that harm the environment, which can create challenges for homestay operators and their communities [31]. This may result in a negative perception of tourists staying at homestays, potentially hindering the sustainability of these accommodations. Convincing local residents of the benefits of tourism is crucial for achieving a more sustainable position.

Transitioning to the social aspect, while tourists contribute positively to the country's revenue, their presence can strain social facilities due to inconveniences and traffic congestion [11]. From an economic perspective, tourists who choose homestays can create job opportunities for the community, directly increasing the income of both the community and homestay operators. However, this positive economic impact can also have adverse effects, such as attracting foreign labor and potentially reducing local employment opportunities [11].

## 5 Methodology

### 5.1 Study area

**Fig. 2:**
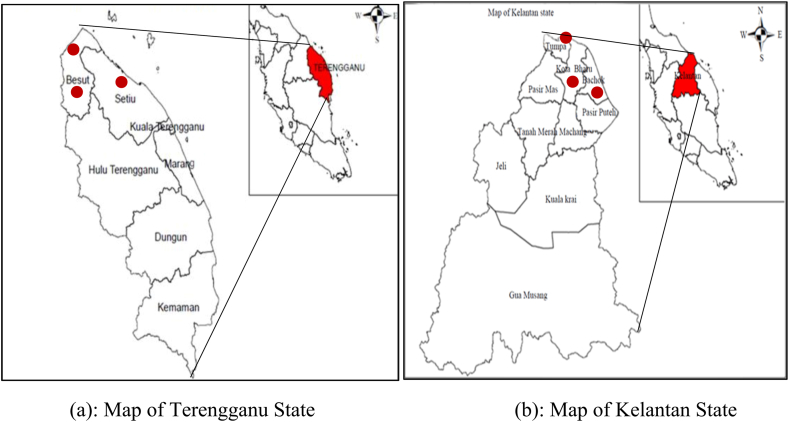
(a) Map of Terengganu state. (b) Map of Kelantan state.

**Fig. 3:**
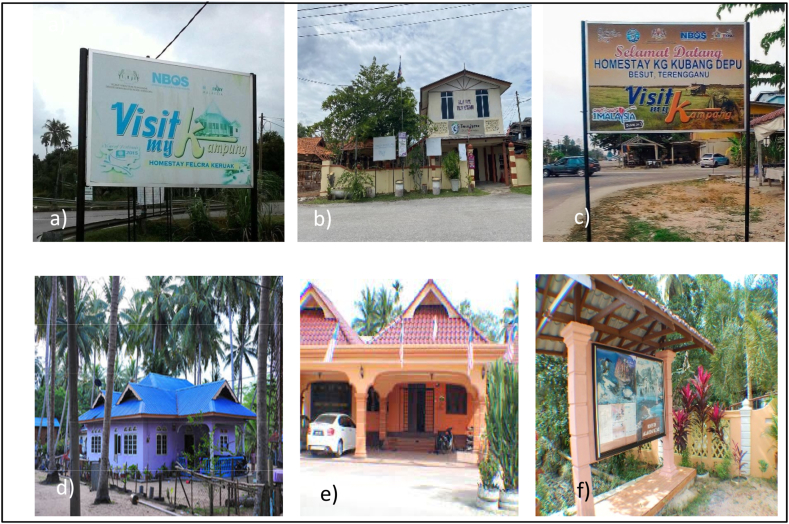
Homestays that have been selected in Terengganu (a, b, c) and Kelantan (d, e, f): **a)** Homestay Felcra Keruak, Jerteh **b)** Homestay Kg. Teluk Ketapang, Kuala Nerus **c)** Homestay Kg. Kubang Depu, Jerteh **d)** Homestay Nelayan Pantai Suri, Tumpat **e)** Homestay GDW Seterpa, Kota Bharu **f)** Homestay Kg. Kubang Telaga, Bachok [All these photo were taken by Fatin Amirah binti Zamzuki and she is the first author of this paper].

This study was conducted on the East Coast of Peninsular Malaysia, specifically in the states of Terengganu (Fig. 2(a)) and Kelantan (Fig. 2(b)). From a total of 35 registered homestays located in the East Coast region of Peninsular Malaysia [5], we selected six homestays for our study. In Terengganu, we selected three homestays (Fig. 3(a), (b), and (c)), and in Kelantan, we chose three homestays (Fig. 3(d), (e), and (f)). These study sites were chosen due to their significant potential to become prominent tourist destinations within Malaysia.

### 5.2 Delphi method

The Delphi method [47,48] was employed to systematically gather information, construct a model, and gather informed judgments from a panel of experts for evaluating the sustainability of homestays. This iterative process can be repeated in multiple rounds until a consensus is reached. Given the absence of definitive answers to comprehensively analyze every aspect, achieving consensus serves as a valuable and acceptable alternative. The Delphi Method was initially developed by Dalkey and Helmer at the Rand Corporation in the 1950s [49]. What sets the Delphi method apart is its approach to eliciting and refining group judgments, founded on the principle that a group of experts can outperform a single expert when precise knowledge is lacking. The Delphi procedure begins by assembling a small group of individuals with expertise in a particular area. They are then presented with queries designed to simulate potential future scenarios within their field of expertise. It's important to note that while these individuals are collectively referred to as a panel, they typically have limited or no prior familiarity with each other.

In the context of assessing homestay sustainability, the Delphi method was selected due to its ability to provide the most appropriate and insightful alternatives, particularly through the input of experts closely associated with homestays. In essence, their direct participation in the survey allowed for a comprehensive evaluation of homestay sustainability, with a focus on the primary factors influencing the industry's future sustainability. This method operates as a predictive framework involving several rounds of questionnaires distributed to a panel of experts [50]. After each round of questionnaires, experts receive a summary of the previous round's responses, enabling them to adjust their answers in alignment with the group's feedback. The Delphi technique combines the strengths of expert analysis with a collaborative element, making it a valuable research methodology, especially in scenarios where definitive or knowable answers are lacking, such as decision-making, policy formulation, or long-term forecasting.

In addition to its traditional use as a forecasting tool, the Delphi procedure is highly adaptable and capable of generating insights and consensus on a wide range of subjects [49,50]. In the context of this study, the presented findings emerged from two rounds of Delphi surveys conducted to gather expert opinions on designing measures for assessing the progress of tourism products towards higher or lower sustainability rankings [11]. Moreover, this research is part of a broader initiative aimed at developing indicators that individuals can use to make more informed vacation choices and contribute to a more sustainable tourism industry [48].

The outcomes of this expert study reveal a significant level of disagreement regarding the definition of “sustainability” and the delineation of its conceptual boundaries. Additionally, the research highlights varying perspectives on the utilization of qualitative and quantitative metrics and the roles each should play in sustainability assessment. It's noteworthy that, despite its versatility, the Delphi technique has not previously been employed to evaluate the sustainability of homestays in Malaysia.

### 5.3 Questionnaire development

For this study, we prepared and distributed 51 sets of questionnaires to a panel of experts, covering three aspects: resources, community, and tourism. These aspects were chosen based on specific criteria. The experts included resource administrators, who represented the resource aspect; homestay operators, who represented the community aspect; and staff from the Ministry of Tourism, Arts, and Culture (MOTAC), who represented the tourism aspect.

The questionnaire set consisted of 42 indicators designed to measure the factors influencing the sustainability of homestays. A 5-point *Likert scale* [11] was employed in the questionnaire, where ‘strongly agree’ was rated as 5, ‘agree’ as 4, ‘neutral’ as 3, ‘disagree’ as 2, and ‘strongly disagree’ as 1. Due to the COVID-19 epidemic, the questionnaires were distributed via email to avoid face-to-face interviews.

To evaluate the sustainability of the homestays, it was crucial to test the validity and reliability of each question intended for use in the assessment. Questions were deemed valid and reliable if they produced a Cronbach's Alpha value exceeding 0.6; otherwise, they were considered invalid or unreliable, signifying potential inconsistencies or inaccuracies. Therefore, prior to distribution in a real homestay environment, the questionnaire underwent a pilot study to ensure its effectiveness.

In its initial development, the questionnaire comprised 42 indicators. However, for questions to achieve the desired levels of validity and reliability (Cronbach's Alpha score >0.6), a screening process was initiated. Questions that fell short of this threshold, indicating inconsistency or imprecision, were removed. As a result, the final questionnaire, used in both rounds of data collection, consisted of 41 indicators.

In the questionnaire distributed during both rounds, respondents were presented with five answer options: ‘*strongly agree*,’ ‘a*gree*,’ ‘*neutral*,’ ‘*disagree*,’ and *‘strongly disagree*.

### 5.4 Selection of experts

To determine the eligibility of potential panelists, specific criteria were established. These criteria included a requirement for panel members to be actively engaged in resource management and to possess a minimum of three years of experience in this field. In the community sector, panelists were expected to have significant experience overseeing homestays and to have undergone substantial training, thereby gaining expertise within the homestay industry. For the tourism domain, panelists were required to have collaborated with tourism agencies or MOTAC, thereby gaining experience in the tourism sector. Additionally, they should have firsthand experience staying at homestays.

All selected panel members met the established criteria for resource administrators, homestay operators with ample work experience or knowledge in the field, and individuals employed in organizations connected to the homestay industry. As a result, the chosen panel members possessed the qualifications necessary to provide the essential information required to achieve the objectives of study.

### 5.5 Procedure of Delphi method for homestay sustainability

This study employed the Delphi technique, which involved two rounds of engagement with experts who were recommended [47,48,51]. In the first round, an open-ended questionnaire was used, employing a qualitative method. Due to COVID-19, the questionnaire was distributed to respondents via email, enabling experts to express their opinions about specific fields by analyzing the content provided. In the second round, the questionnaire was expanded based on the responses received in the first round. This phase included quantitative questions, and an effort was made to seek expert consensus. Furthermore, statistical techniques were applied to analyze the data from this second questionnaire. The Delphi procedure for the first and second rounds and the research process employed in this study are illustrated in Fig. 4(a), Fig. 4(b)(a) and 4(b).

**Fig. 4(a):**
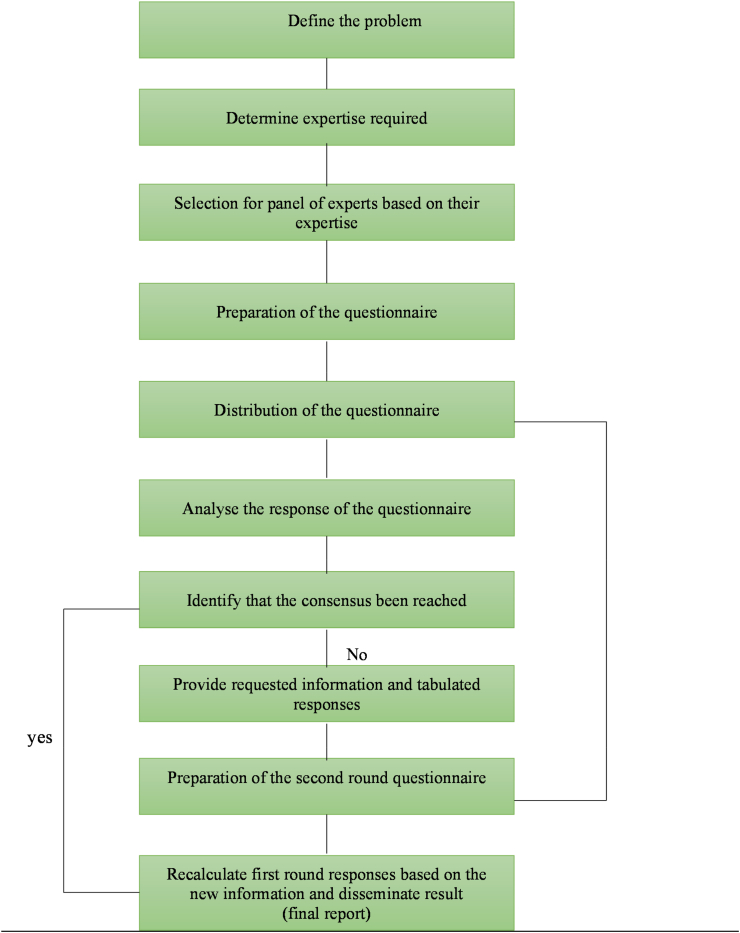
The procedure in Delphi Method of homestay sustainable tourism development.

**Fig. 4(b):**
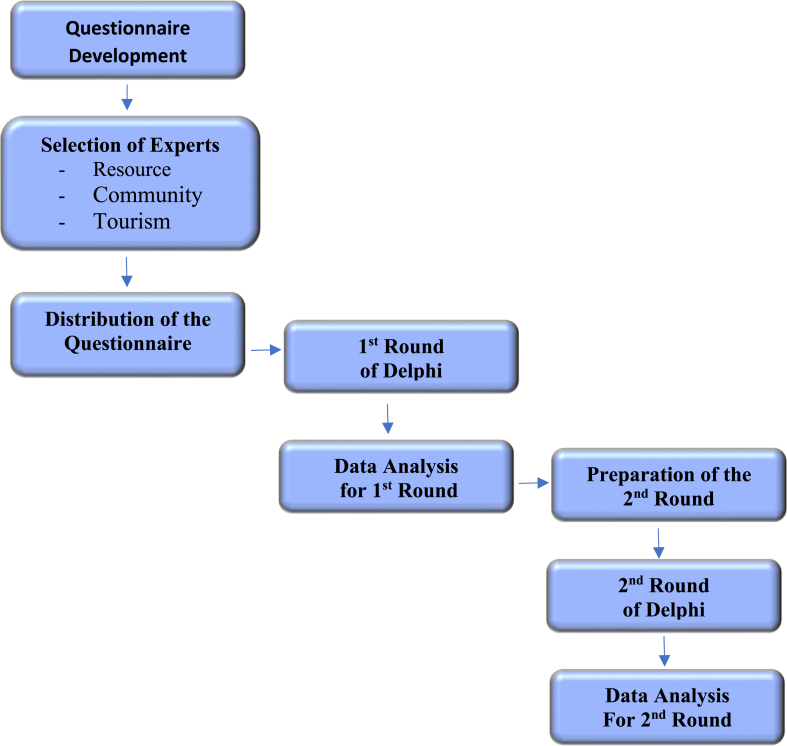
The flowchart of the research process of homestay sustainable tourism development.

#### 5.5.1 First round of Delphi

The first round in the Delphi method is crucial and serves the purpose of identification. During this phase, each expert was asked to list coordinating variables within their field based on their knowledge. They were also instructed to exclude irrelevant variables. Following the first round of interviews, the collected data were meticulously analyzed, resulting in the compilation of a coordination list.

The initial round of the Delphi method took place in January 2020, involving 51 sets of questionnaires distributed to the panel, covering three aspects: resources, community, and tourism. The questionnaires were disseminated via email and returned after all the questions had been answered.

In this round, the survey results underwent a Cronbach's Alpha test, similar to the process during questionnaire development. During this test, each question's validity and reliability, as obtained from the panel, were assessed using Cronbach's Alpha. Any question with a Cronbach's Alpha value below 0.6 was excluded. Consequently, the number of questionnaire indicators was reduced to 41 after this deletion process, and these remaining indicators were employed in the second round.

#### 5.5.2 Second round of Delphi

At this stage, the experts were requested to re-evaluate their assessments based on the results gathered in the first round. The raw data obtained were analyzed using SPSS software. Subsequently, Kendall's coefficient of concordance (W) was calculated to determine whether the respondents ranked the adjustment variables in the same order. A concordance coefficient of 1 indicates unanimous ranking among all respondents, while a coefficient of 0 signifies that respondents assigned different rankings to the variables.

The second round of the study commenced in February 2020, with the content of this survey remaining consistent with the first round, except for the number of indicators. The results from the initial round were distributed to the respondents. In this round, 51 sets of questionnaires were returned from the experts, and the *t*-test was employed to determine whether the opinions expressed in the first and second rounds were consistent. The significance level used was α = 0.05, and the p-value for all indicators exceeded 0.05. The study revealed a slight improvement in the second round compared to the first, although the mean score remained relatively unchanged. Consequently, it is anticipated that the next round of research may not yield any significant shifts in expert opinions, as all participants have already expressed their agreement and reached a consensus.

### 5.6 Sustainability homestay indicator

The assessment of homestay sustainability relies on stakeholders' perceptions and their interactions. The average mean score serves as the basis for determining the homestay's sustainability level. Overall sustainability is computed using sustainability ratings from six distinct relationship-related factors. Formulas are applied to ascertain the weight, weighted score, and percentage of achievement (100%). In this study, Allen's Sustainability Barometer was adopted, and additional indicators for the mean score were included to bolster the homestay industry's sustainability assessment, as outlined in Table 1. The indicator's mean score scale ranges from 1 to 5, where 1.0–2.0 signifies “unsustainable,” 2.1–3.0 indicates “potentially unsustainable,” 3.1–4.0 represents “potentially sustainable,” and 4.1–5.0 denotes “sustainable.” Regarding the analysis of the outcomes, the approach encompasses (1) presenting the percentage achievement results (%) using a spider web diagram, with the diagram's axes depicting the percentage achievement for each interconnected aspect, and (2) categorizing the evaluation results into sustainable and unsustainable categories based on an interval scale.

**Table 1:** Table of barometer scale: the degree of sustainability.

Sector	Scores on scale	Indicators mean score
Unsustainable	0≤γ<25	1.0−2.0
Potentially unsustainable	25≤γ<50	2.1−3.0
Potentially sustainable	50≤γ<75	3.1−4.0
Sustainable	75≤γ≤100	4.1−5.0

### 5.7 Analysis of data

To evaluate the sustainability of homestays, we employed the mean score, weights, and score weighting indicators proposed by Tsaur et al. [11]. The weights, which reflect the importance of each indicator, are calculated using the following formula.

(1) $$\varepsilon_{ij}=\left(\frac{\alpha }{\beta }\right)\times 100$$

where $\left(\varepsilon_{ij}\right)$ is the indicator weight, while α and *β* are the mean score and total mean score of each indicator, respectively.

Next, the weighted score on the indicators related to the sustainability aspect of the homestay is calculated using the following equation.

(2) $$\sigma_{ij}=\left(\frac{\lambda -1}{4}\right)\times \varepsilon_{ij}$$

where $\left(\sigma_{ij}\right)$ is the weighted score and λ is the score of the indicator.

In order to determine the sustainability of the homestay, the sustainability rating of six different relationship-related factors was calculated based on the following equation.

(3) $$\sigma_{i}=\sum_{j=1}^{n}\sigma_{ij}$$

where $\sigma_{i}$ and *n* denotes the sum of the weighted scores $\left(\sigma_{ij}\right)$ for the i ^th^ and the number of indicators, respectively.

$$\sigma =\sum_{i=1}^{6}\sigma_{i}$$

σ is denoted as the sum of the weighted scores for the tourism development system. Finally, to measure the contribution of a sustainable homestay from the relationship aspect, the achievement score is calculated using the following equation proposed by Tsaur et al. [11].

(4) $$F_{i}=\frac{\sigma_{i}}{\varepsilon_{i}}\times 100\%$$

where $F_{i}$ and $\varepsilon_{i}$ denoted as the i ^th^ relationship aspect and the sum of weight, respectively.

## 6 Empirical evaluation of homestay sustainability

### 6.1 Evaluation results

Based on the relationship framework involving resources, the local community (homestay operator), and tourism (tourist), this study aims to assess the sustainability of homestay tourism from the perspective of stakeholders. In essence, it seeks to evaluate the impact of homestay sustainability through the following perspectives:

1. The local community (LC) serves as the subject for assessing the influence of tourism and resources on the community.
2. Resource administrators (RA) are the focus when evaluating the effect of tourism and the community on resources.
3. Tourists are considered subjects when assessing the impact of resources and the community on tourism."

**Fig. 5:**
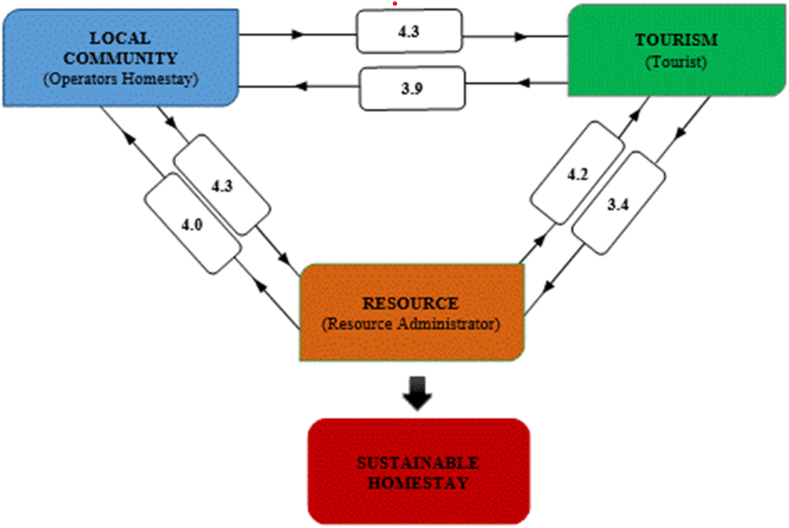
Score of homestay sustainable industry for the East Coast of using the Delphi Method.

To assess homestay sustainability, we adopted the Sustainability Barometer scale as proposed by Ref. [52] and enhanced it with a mean score to strengthen the evaluation of homestay sustainability. The assessment results, which reflect the degree of sustainability for all indicators within the relationship aspect, are presented in Fig. 5. The “Influence of Community on Tourism” indicator performed the best with a mean score of 4.34, falling within the “sustainable interval.” This suggests a sustainable relationship between tourists and the local community (LC). Conversely, the “Influence of Tourism on Resources” indicator performed the lowest with a mean score of 3.62, falling within the “potential sustainable interval.” Despite being the lowest-rated among the relationship aspects, it still holds the potential for sustainability, as tourism successfully generated employment opportunities and economic benefits for the local community.

Further discussion of all relationship aspects is provided in Table 2. According to this table, the tourism community maintains the highest position with a weight of 30.02, while community resources have the lowest weight at 8.15. The remaining aspects include community resources (25.79), tourism resources (14.28), resource tourism (13.42), and community tourism (8.35). These results align with the findings of Tsaur et al. [11], who also observed that tourism communities received the highest scores, while community resources received the lowest scores. They emphasized the importance of basing tourism development on the sustainable use of resources. Negative impacts on resources not only endanger community sustainability but also diminish the appeal of the destination.

**Table 2:** Sustainable homestay indicators system and weight.

Relationship Aspect	Weight	Dimension	Weight	Indicator	Weight
Resource Community	25.8	Economics	2.7	1. Increase the income of the rural community	2.9
				2. Open new job opportunities to the rural community	2.7
				3. Improving economy	2.7
				4. Eliminate rural poverty	2.7
				5. Improve management skills and knowledge	2.6
		Environment	2.6	6. Improve the environment and quality of life	2.6
		Social	2.4	7. Disrupt the daily life of the local community	1.5
				8. Open educational opportunities in management	2.7
				9. Increased awareness of environmental protection	2.7
				10. Establish good interactions between residents, homestay operators and tourists	2.7
Community Resource	8.2	Economics	2.7	11. To generate income that can be contributed to rural development without the use of environmentally harmful materials	2.7
		Environment	2.7	12. Promotes recycling activities	2.7
		Social	2.7	13. The villagers are involved in resource management and planning	2.7
Resource Tourism	13.4	Economics	2.7	14. Upgrading the homes of homestay entrepreneurs and rural infrastructure	2.7
		Environment	2.8	15. Water quality assessments for daily use of homestays are also monitored	2.8
				16. Attract visitors	2.9
		Social	2.5	17. The resource management section provides quality and enjoyable recreation 18. Tourism development is very satisfying	2.6
					2.5
Tourism Resource	14.3	Economics	2.7	19. Provides economic contribution to conservation	2.7
		Environment	1.7	20. The natural resources can be conserved and conserved	2.7
				21. Provides sustainable and sustainable results to the environment	2.7
				22. Causes water pollution	1.3
				23. Disturbing natural resources	1.3
				24. Destroys environmental quality	1.3
				25. Destroys the local environment	1.3
				26. Destroys air quality in rural areas	1.3
Community Tourism	8.4	Environment	2.8	27. Enhance the image and beauty of the village	2.9
				28. Provides opportunities for tourists to enjoy a clean rural environment	2.8
		Social	2.7	29. Introducing local culture and traditions to tourists	2.7
Tourism Community	30.0	Economics	2.6	30. Attract many villagers to fill their free time and earn extra income by participating in this homestay program	2.6
				31. Improve management experience among homestay entrepreneurs	2.7
				32. State economic planning units provide infrastructure such as jetty construction and other for the benefit of residents such as jetty construction etc.	2.3
				33. Reducing the income gap between homestay entrepreneurs and tourists	2.5
				34. Encourages the local handicraft industry	2.8
				35. Proceeds from tourism are distributed for rural development	2.6
		Environment	2.6	36. Waste management is also enhanced by the existence of homestays	2.6
		Social	2.4	37. Reduce crime and social symptoms in rural areas	2.7
				38. Increase local cultural awareness among residents	2.6
				39. Opening opportunities for cultural exchange between the local community and tourists	2.6
				40. Creating a united society	2.6
				41. Causing traffic congestion at peak hours	1.4

#### 6.1.1 Influence of community on tourism

The ‘Influence of community on tourism’ scored the highest at 4.34, as shown in Fig. 5, indicating ‘sustainability.’ Based on Table 3, a score of 4.45 for the environment dimension demonstrates a strong inter-relationship. Local communities, who are homestay operators, take the initiative to improve the image and beauty of the village to attract tourists to stay at the homestay. Furthermore, homestay tourism has opened up opportunities for tourists to enjoy a clean rural environment.

**Table 3:** Weighted score of homestay sustainable tourism development.

Relationship Aspect	Dimension	Mean score	Indicator	Score	Weighted score
Resource Community	Economics	4.3	1. Increase the income of the rural community	4.6	2.6
			2. Open new job opportunities to the rural community	4.3	2.2
			3. Improving economy	4.3	2.3
			4. Eliminate rural poverty	4.3	2.2
			5. Improve management skills and knowledge	4.2	2.1
	Environment	4.1	6. Improve the environment and quality of life	4.1	2.0
	Social	3.7	7. Disrupt the daily life of the local community	2.3	0.5
			8. Open educational opportunities in management	4.2	2.1
			9. Increased awareness of environmental protection	4.2	2.1
			10. Establish good interactions between residents, homestay operators and tourists	4.3	2.2
Community Resource	Economics	4.3	11. To generate income that can be contributed to rural development without the use of environmentally harmful materials	4.3	2.3
	Environment	4.3	12. Promotes recycling activities	4.3	2.3
	Social	4.2	13. The villagers are involved in resource management and planning	4.2	2.2
Resource Tourism	Economics	4.3	14. Upgrading the homes of homestay entrepreneurs and rural infrastructure	4.3	2.2
	Environment	4.4	15. Water quality assessments for daily use of homestays are also monitored	4.4	2.3
			16. Attract visitors	4.5	2.5
Tourism Resource	Social	4.0	17. The resource management section provides quality and enjoyable recreation	4.1	2.0
			18. Tourism development is very satisfying	3.9	1.8
	Economics	4.3	19. Provides economic contribution to conservation	4.3	2.2
	Environment	2.6	20. The natural resources can be conserved and conserved	4.2	2.1
			21. Provides sustainable and sustainable results to the environment	4.2	2.1
			22. Causes water pollution	1.9	0.3
			23. Disturbing natural resources	2.0	0.3
			24. Destroys environmental quality	2.0	0.1
			25. Destroys the local environment	2.0	0.3
			26. Destroys air quality in rural areas	2.0	0.3
Community Tourism	Environment	4.5	27. Enhance the image and beauty of the village	4.5	2.5
			28. Provides opportunities for tourists to enjoy a clean rural environment	4.4	2.3
	Social	4.2	29. Introducing local culture and traditions to tourists	4.2	2.2
Tourism Community	Economics	4.1	30. Attract many villagers to fill their free time and earn extra income by participating in this homestay program	4.0	2.0
			31. Improve management experience among homestay entrepreneurs	4.2	2.2
			32. State economic planning units provide infrastructure such as jetty construction and other for the benefit of residents such as jetty construction etc.	3.6	1.5
			33. Reducing the income gap between homestay entrepreneurs and tourists	4.0	1.9
			34. Encourages the local handicraft industry proceeds from tourism are distributed for rural development	4.5	2.5
			35. Proceeds from tourism are distributed for rural development	4.1	2.0
	Environment	4.2	36. Waste management is also enhanced by the existence of homestays	4.2	2.1
	Social	3.8	37. Reduce crime and social symptoms in rural areas	4.2	2.1
			38. Increase local cultural awareness among residents	4.1	2.0
			39. Opening opportunities for cultural exchange between the local community and tourists	4.1	2.0
			40. Creating a united society	4.1	2.0
			41. Causing traffic congestion at peak hours	2.3	0.5

Additionally, the social dimension scored 4.24, indicating that efforts by local communities have a positive inter-relationship. This implies that by introducing local culture and traditions to tourists, tourists can get to know and learn about new cultures and traditions.

#### 6.1.2 Influence of community on resources

The community resource factor received the second-highest mean score at 4.27 (Fig. 5), falling within the ‘sustainability’ range. According to Table 3, the economic dimension shows a score of 4.29, indicating that most of the communities generating income in this industry contribute to rural development without harming the environment. Furthermore, the environmental dimension scored 4.31, indicating that the local community around the homestay promotes recycling activities. This helps minimize waste disposal, air pollution from the combustion process, and water pollution. The social dimension also scored among the highest at 4.22, showing that the residents around the homestay are actively involved in management and planning.

#### 6.1.3 Influence of resources on tourism

The category “Influence of resources on tourism” obtained a score of 4.34 (as depicted in Fig. 5), signifying a state of “sustainability.” Meanwhile, Table 3 showed that the “economic dimension” achieved a score of 4.25. This implies that the economic measures undertaken by the resource sector, such as enhancing rural lodgings and internal infrastructure, have yielded positive outcomes. Additionally, the “environmental dimension” garnered a score of 4.43, indicating that resource management's water quality efforts have been effective. Proficient resource management holds the potential to attract tourists to choose homestays, fostering an appreciation for the environment.

#### 6.1.4 Influence of resources on community

The nature of the ‘Influence of resources on the community’ showed a positive influence with a score of 4.05 (Fig. 5), indicating ‘sustainability’ concerning the local community. According to Table 3, the economic dimension score for this factor is 4.31, indicating that resource administrators play a crucial role in homestay sustainability. They can create job opportunities and increase rural income, thus alleviating rural poverty.

Additionally, the environmental dimension for this factor scored 4.09, suggesting that resource administrators can improve the quality of the environment around the homestay. Furthermore, the social dimension scored 3.74, representing a high level of interaction: resource administrators exhibit positive interactions with homestay and tourism operators. In other words, residents are willing to support resource conservation and participate in resource administration activities, contributing to the well-being of rural communities and the growth of the homestay business.

#### 6.1.5 Influence of tourism on community

Table 3 and Fig. 5 reveal that the mean score for the ‘Influence of tourism on the community’ is 3.99, signifying a positive impact of tourism on the community. This score suggests that tourists have engaged with the local community in a positive manner, fostering goodwill and interaction. Tourism plays a pivotal role in homestays, particularly in economic activities, as it provides income and employment opportunities for the local community, significantly contributing to regional growth.

The economic score of 4.06 demonstrates that domestic tourism can generate profits for local entrepreneurs. From a community perspective, the expansion of homestay tourism enhances the management skills of homestay operators while creating job opportunities and additional income for the rural community. Moreover, the environmental score of 4.16 indicates that homestay tourism contributes to improved waste management practices. Furthermore, homestays facilitate cultural exchange between the rural community and tourists, allowing for the introduction and promotion of local culture on an international scale.

#### 6.1.6 Influence of tourism on resources

Fig. 5 reveals that the influence of tourism on resource factors recorded the lowest score among all interaction relationships, with a score of 3.62. However, this score is still considered very positive and falls within the range of ‘potentially sustainable.’ The economic dimension, with a mean score of 4.25, demonstrates that tourism's impact on resources can provide an economic contribution to resource conservation. Therefore, the existence of homestays can contribute to the proper maintenance and preservation of resources.

However, the environmental dimension for the influence of tourism on resources recorded the lowest score at 2.60 (Table 3), falling into the category of ‘potentially unsustainable.’ The influx of a large number of tourists has led to the deterioration of the natural resource environment's quality during their homestay vacations. The analysis (Table 3) reveals that an apparent excessive number of tourists has already caused a negative impact on resources, particularly in terms of water pollution, disruption of natural resources, degradation of environmental quality in the local area, and poor air quality in rural areas. Therefore, resource management should take appropriate action to prevent such problems from occurring.

#### 6.1.7 Overall evaluation of homestay sustainability

**Fig. 6:**
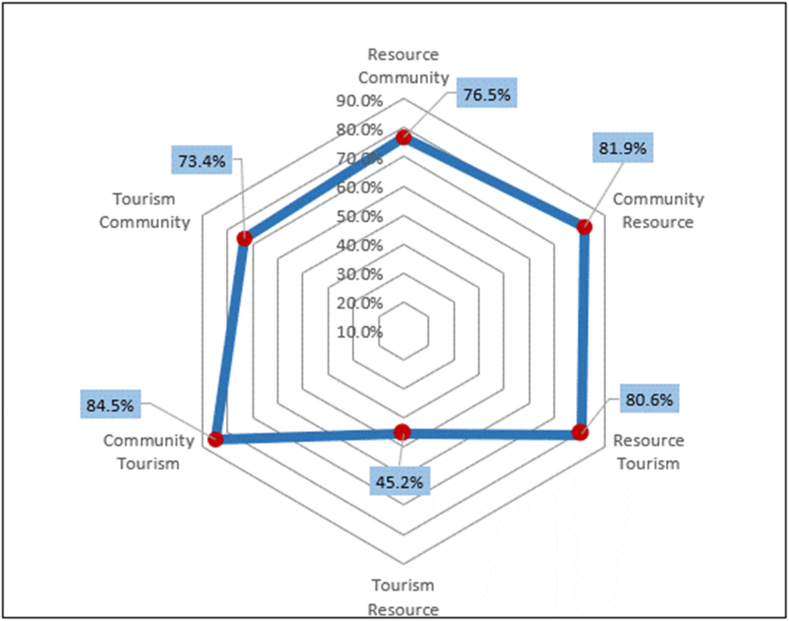
Sustainability achievement (%) of homestay in inter-relationship aspects.

To ascertain the comprehensive sustainability of the homestay, an overall sustainability score encompassing six interrelated aspects was utilized. The weight, weighted score, and attainment percentage (100%) were computed using Equations (2), (3), with reference to Table 4. As indicated by Fig. 6, the highest impact was observed in the “influence of community on tourism” with a score of 84.5%, followed by the “influence of community on resource” at 81.9%, while the lowest sustainability was associated with the “influence of tourism on the community” with a score of 45.2% The homestay's weighted sustainability scores, linked to the achievement of sustainability across interconnected resources, fall within the “sustainable” classification.

**Table 4:** Sustainability achievement of homestay in inter-relationship aspects.

Sources	Weight	Weighted score	Achievement percentage (%)
Resource Community	25.7	20.3	76.5
Community Resource	8.2	6.7	81.9
Resource Tourism	13.4	10.8	80.6
Tourism Resource	14.3	7.9	45.2
Community Tourism	8.4	7.1	84.5
Tourism Community	30.0	22.6	73.4
**Total**	**100.0**	**75.4**	**442.1**

In this section, we address three research questions concerning the sustainability of homestays in the East Coast states of Kelantan and Terengganu, focusing on the influence of resources, the local community, and tourism on homestay sustainability. Our findings reveal positive interactions among these variables, underscoring the potential for sustainable homestays. However, we emphasize the importance of ongoing stakeholder collaboration and the prevention of inappropriate decisions to ensure sustainability.

This study identifies three primary factors influencing host family sustainability: resources, the local community, and tourism. Our evaluation of these factors unveils various aspects of their influence on each other. For instance, the influence of the community on tourism emerges as the strongest, while the potential for improvement exists regarding the influence of tourism on resources. These findings underscore the significance of fostering positive interactions between these factors to achieve sustainable homestays.

While our research doesn't directly address alternative methods for enhancing homestay sustainability without impacting the natural ecosystem, it highlights the adverse effects of tourism on the environment, particularly in terms of water pollution and natural resource degradation. We suggest that appropriate resource management measures should be implemented to address these issues.

Our findings emphasize the potential role of sustainable practices and responsible tourism strategies in enhancing homestay sustainability while reducing negative impacts on the natural ecosystem. As we delve into the sustainability of homestays, our study indirectly prompts questions about alternative approaches to enhance sustainability without compromising the natural ecosystem. Ultimately, our research underscores the importance of resource management actions and responsible tourism practices to mitigate negative impacts and improve sustainability outcomes.

## 7 Conclusion, research limitations and future directions

The homestay industry serves as a catalyst for improving the socioeconomic status of rural populations through tourism. It presents a highly profitable investment opportunity for rural communities and holds significant potential as a long-term strategy to enhance Malaysia's tourism image on the global stage. In this study, we have developed and validated a novel assessment method for evaluating the sustainability of the homestay sector in the eastern coastal states of Kelantan and Terengganu. Our investigation focused on three key variables that exert influence on homestay sustainability: resources, the local community, and tourism. Within these variables, we identified six distinct relationships. Our findings indicate that the influence of the local community is the most significant and falls within the “sustainable” category, while the community's influence on resources is the least significant.

When considering these relational aspects, the influence of the community on both resources and tourism received the highest percentage. Conversely, tourism's impact on resources received the lowest mean score, categorizing it as “unsustainable.” It is crucial to recognize that fostering positive interactions between resources, the local community, and tourism is of paramount importance for any homestay striving for sustainability. Effective management should continually assess stakeholder collaboration and take measures to prevent inappropriate decisions that may disrupt the balance among these stakeholders and their relationships.

Moreover, the homestay environment provides an excellent opportunity to nurture the growth of the local handicraft industry, ensuring its sustainability in the years to come. Developing authentic handicrafts that reflect the rich local culture and heritage has the potential to significantly boost tourist attraction and invigorate the broader tourism sector. Instead of solely relying on government initiatives, introducing handicraft activities within homestays can effectively attract both domestic and international tourists, enticing them to visit and extend their stays. Consequently, handicrafts that authentically represent local culture and heritage can serve as catalysts for heightened tourist interest, propelling the advancement of the tourism sector. This innovative approach could be expanded further to explore comparisons between peninsular Malaysia and the Malaysian Borneo states. By encompassing a comprehensive blend of extrinsic and intrinsic factors, it holds the potential to formulate a holistic model contributing to the overall development of the country's tourism industry.

However, it is important to acknowledge several limitations encountered during this study, which can provide valuable guidance for future research. Firstly, this study employed ‘purposive sampling’ and specific criteria to assess the sustainability of homestays in the states of Kelantan and Terengganu. This approach may limit the generalizability of the study's findings to the entire homestay population. To validate the applicability of the developed method for assessing homestay sustainability, future research should consider extending the study to other states with similar homestay characteristics.

Secondly, there is a noticeable gap in research concerning the knowledge of local communities regarding the sustainability of homestays in developing countries and their perceptions of the associated benefits and costs. Much of the existing research on homestays, particularly from a socio-cultural perspective, has primarily focused on Western societies. In these societies, local communities are generally more aware of tourism and its impacts on their perceptions of costs and benefits. Hence, it is crucial to explore the level of knowledge about tourism, especially in developing countries where it may differ significantly from that in developed nations. Furthermore, it is important to recognize that local communities in these developing regions may hold distinct perceptions regarding the sustainability of homestay development.

Thirdly, this study focused exclusively on factors influencing resilience, such as community participation, community culture, relationships with authorities, community flexibility, and environmental conditions. It did not consider additional factors encompassing legal, regulatory, and partnership aspects, including professional attitudes, lack of expertise, elite dominance, absence of an appropriate legal framework, insufficient trained human resources, relatively high costs associated with community participation, and inadequate financial resources. Future research should actively investigate these factors to gain a deeper understanding of their impact on homestay sustainability.

While this study primarily centered on host families for its empirical investigation, there is potential for future research to explore how the Delphi assessment of sustainability factors can be applied to other areas such as natural and protected areas, ecotourism, cultural heritage, wildlife, and more. It is advisable to include specific types of homestay programs like CBT, CBET, CBAT, and additional indicators to enhance the evaluation process. Although the results of this study revealed significant influences of the community and tourism on the environment, it's apparent that the selected indicators may be insufficient. Therefore, it is recommended to consider incorporating additional indicators in future research. Furthermore, for upcoming studies, providing Delphi experts with more detailed information can lead to a better understanding of the real situation and facilitate the refinement of indicator definitions.

Empirical findings can serve as valuable tools in assisting local communities with effective management strategies. A deeper understanding of the challenges faced by local communities and their corresponding actions can be instrumental in reshaping the homestay sector. Consequently, we can redefine and revitalize the homestay industry with a ‘community-centered homestay framework.’ Within this framework, the process of resource development assumes a pivotal role; that is, the administration should take proactive steps to engage and encourage the active participation of the local community in the planning and recommendation phases. In addition to providing the framework, the administration may also create employment opportunities within the tourism sector for local communities, such as offering interpretation services and showcasing traditional cultural and craft performances. Consequently, the preservation of the local community's livelihood can be achieved, resulting in a more attractive living environment.

MOTAC launched the ‘Visit Truly Asia Malaysia 2020' campaign, in which homestays were identified as a crucial element for potential tourism development. Many activities organized by homestays, coupled with increased community participation, are gaining popularity. Consequently, numerous homestays are now grappling with the phenomenon of ‘superficially adhering to ecotourism principles while indirectly harming the environment.’ Questions regarding the preservation of fair community interactions, the potential environmental impact, and the sustainability of natural resources have not received the attention they deserve. From a practical standpoint, resource management, the local community, and tourists all face pressing challenges that demand proactive solutions.

The role of social media platforms, such as Facebook, Twitter, Telegram, and others, should be considered a primary factor in assessing social indicators when researching the future sustainability of homestays. Social media has supplanted traditional media channels like TV and newspapers in rapidly delivering and disseminating information to communities. It has become the predominant channel for information dissemination, representing a pervasive trend across all segments of society due to its efficiency in conveying information. Therefore, all stakeholders in the homestay industry, particularly operators, should fully leverage the advantages of social media to ensure the future sustainability of homestays.

## CRediT authorship contribution statement

**Fatin Amira Zamzuki:** Conceptualization, Data curation, Formal analysis, Methodology, Writing – original draft. **Muhamad Safiih Lola:** Conceptualization, Formal analysis, Funding acquisition, Investigation, Methodology, Project administration, Supervision, Validation, Writing – original draft, Writing – review & editing. **Elayaraja Aruchunan:** Conceptualization, Funding acquisition, Validation, Writing – review & editing. **Mohana Sundaram Muthuvalu:** Funding acquisition, Writing – review & editing. **Ribed Vianneca W. Jubilee:** Funding acquisition, Writing – review & editing. **Nurul Hila Zainuddin:** Funding acquisition, Methodology, Validation. **Abd. Aziz K. Abd Hamid:** Funding acquisition, Software, Validation, Visualization. **Nor Aieni Mokhtar:** Conceptualization, Funding acquisition, Validation, Writing – review & editing. **Mohd Tajuddin Abdullah:** Conceptualization, Investigation, Writing – review & editing, Funding acquisition, Validation, Writing – original draft.

## Declaration of competing interest

The authors declare the following financial interests/personal relationships which may be considered as potential competing interests:Mohd Tajuddin Abdullah reports financial support was provided by the MNRECC-DWNP Giant Panda Protection and Research Programme. Mohana Sundaram Muthuvalu reports financial support was provided by Universiti Teknologi PETRONAS-Universiti Malaysia Pahang Matching Grant.
